# Collaboration Leading to Addiction Treatment and Recovery from Other Stresses (CLARO): process of adapting collaborative care for co-occurring opioid use and mental disorders

**DOI:** 10.1186/s13722-022-00302-9

**Published:** 2022-04-08

**Authors:** Karen Chan Osilla, Alex R. Dopp, Katherine E. Watkins, Venice Ceballos, Brian Hurley, Lisa S. Meredith, Isabel Leamon, Vanessa Jacobsohn, Miriam Komaromy

**Affiliations:** 1 RAND Corporation, 1776 Main Street, Santa Monica, CA 90401 USA; 2 Department of Psychiatry and Behavioral Sciences, Stanford University School of Medicine, 401 Quarry Road, Palo Alto, CA 94305 USA; 3 Office for Community Health, University of New Mexico Health Sciences Center, , 1650 University Blvd. NE, Suite 3300, Albuquerque, NM 87131 USA; 4 Los Angeles County Department of Health, 1000 S. Fremont Ave, Los Angeles, CA 91803 USA; 5 Department of Psychiatry and Behavioral Sciences, Addictions Division, University of New Mexico Health Sciences Center, School of Medicine, 2400 Tucker Ave NE, Albuquerque, NM 87131 USA; 6 Boston Medical Center, Grayken Center for Addiction, One Boston Medical Center Place, Boston, MA 02118 USA

**Keywords:** Opioid use disorder, Depression, Post-traumatic stress disorder, Collaborative care, Primary care behavioral health, Adaptation, Community health workers

## Abstract

**Background:**

Opioid use disorders (OUD), co-occurring with either depression and/or PTSD, are prevalent, burdensome, and often receive little or low-quality care. Collaborative care is a service delivery intervention that uses a team-based model to improve treatment access, quality, and outcomes in primary care patients, but has not been evaluated for co-occurring OUD and mental health disorders. To address this treatment and quality gap, we adapted collaborative care for co-occurring OUD and mental health disorders.

**Methods:**

Our adapted model is called Collaboration Leading to Addiction Treatment and Recovery from Other Stresses (CLARO). We used the five-step Map of Adaptation Process (McKleroy in AIDS Educ Prev 18:59–73, 2006) to develop the model. For each step, our stakeholder team of research and clinical experts, primary care partners, and patients provided input into adaptation processes (e.g., adaptation team meetings, clinic partner feedback, patient interviews and beta-testing). To document each adaptation and our decision-making process, we used the Framework for Reporting Adaptations and Modifications-Enhanced (Wiltsey Stirman in Implement Sci 14:1–10, 2019).

**Results:**

We documented 12 planned fidelity-consistent adaptations to collaborative care, including a mix of content, context, and training/evaluation modifications intended to improve fit with the patient population (co-occurring disorders) or the New Mexico setting (low-resource clinics in health professional shortage areas). Examples of documented adaptations include use of community health workers as care coordinators; an expanded consultant team to support task-shifting to community health workers; modified training protocols for Problem-Solving Therapy and Written Exposure Therapy to incorporate examples of treating patients for depression or PTSD with co-occurring OUD; and having care coordinators screen for patients’ social needs.

**Conclusions:**

We completed the first three steps of the Map of Adaptation Process, resulting in a variety of adaptations that we believe will make collaborative care more acceptable and feasible in treating co-occurring OUD and mental health disorders. Future steps include evaluating the effectiveness of CLARO and documenting reactive and/or planned adaptations to the model that occur during its implementation and delivery.

*Trial registration* NCT04559893, NCT04634279. Registered 08 September 2020, https://clinicaltrials.gov/ct2/show/NCT04559893

Opioid use disorder (OUD) commonly co-occurs with major depressive disorder (MDD) and/or post-traumatic stress disorder (PTSD) [1–9]. When present, mental health co-morbidities are associated with poorer outcomes [5, 6, 8, 10–15] including higher rates of overdose and suicide than those with OUD alone [16–20]. Medication for OUD (MOUD) reduces the risk of suicide attempts, unintentional overdose, and mortality, yet many people with OUD and co-occurring disorders never receive treatment, and 50–80% of those who do initiate MOUD discontinue treatment, often within weeks or months of initiation [21–25]. Psychological treatment for MDD and PTSD is also associated with decreased suicidality and mortality, but access is low, particularly for those with co-occurring disorders [26–28]. Improved service delivery models are needed to engage people with OUD and co-occurring MDD and/or PTSD in evidence-based treatments.

Collaborative care is an innovative service delivery model that improves access to and the quality of behavioral health care in primary care health systems. Primary care is an opportune setting to address OUD co-occurring with MDD and/or PTSD because the prevalence of OUD is high among primary care patients [29, 30]. This setting offers a relatively accessible and unstigmatized opportunity for receiving treatment [31], and recent federal legislation increased coverage for OUD treatment in primary care [32, 33]. The collaborative care model emphasizes five core principles, centered around a care coordinator who acts as a bridge between the patient and their care team [34]: (1) *patient-centered care* in which primary care providers, behavioral health providers, and care coordinators collaborate with the patient to create treatment plans tailored to the patient’s specific goals, values, preferences, and needs; (2) *population-based care* where the care team works together to engage a specific group of patients (e.g., adults who have OUD co-occurring with MDD and/or PTSD) in care, including those who are not improving or who are missing visits; (3) *measurement-based treatment*, where the care coordinator repeatedly measures symptoms and progress toward goals using validated scales, and treatments are adjusted as needed until the patient improves; (4) *evidence-based care* where patients are offered treatments with research evidence that supports their effectiveness for the target conditions (e.g., MOUD), and (5) *accountable care*, which involves structures and incentives that focus on the provision of quality care and clinical outcomes (rather than quantity of care delivered).

Collaborative care offers a promising model to improve care, but has only shown effectiveness in treating each disorder individually. Results from previous clinical trials suggest that collaborative care can be effectively delivered in primary care to meet the needs of patients experiencing MDD [35], PTSD [36], or substance use disorders [37, 38]. However, it is not clear how these results can be generalized to collaborative care for OUD co-occurring with mental health disorders, given that: (a) individuals with co-occurring disorders often have more complex, severe symptoms and more difficulty engaging and staying in treatment [39], and (b) providing care to this population requires specialized training in multiple clinical issues and treatments.

The purpose of the current study [40] was to document the adaptations we made to collaborative care to treat co-occurring OUD, MDD, and PTSD in primary care settings in New Mexico, a state with numerous Health Professional Shortage Areas (HPSA) and one of the highest rates of mortality associated with suicide and overdose [41]. We focus on OUD, MDD, and PTSD because of existing trials showing effectiveness of collaborative care with these individual conditions. We anticipated that we would need to make adaptations to the care coordinator role (i.e., their qualifications and scope of work), scales used in measurement-based care, and that the types of evidence-based treatments would need to be adapted to provide effective treatment.

This paper describes and documents our adaptation processes, drawing on approaches from implementation science, a rapidly growing field studying the processes by which practice settings adopt evidence-based practices into routine health care [42]. Adaptations are planned modifications to intervention design or delivery to improve effectiveness or fit with certain populations—e.g., clinical problems, sociocultural characteristics—or conditions—e.g., providers, settings [2]. Historically, implementation researchers have emphasized maintaining fidelity to the original practice, but in recent years have recognized that thoughtful adaptation is important to the success of any new application of an evidence-based practice [1, 43, 44]. Documenting adaptation processes and outcomes is important because it captures key information about the adapted intervention, which is necessary for replication, interpretation of study findings and the potential for transportability of the intervention to new settings. Our research questions were: (1) what adaptations were made to the collaborative care model for use with OUD and co-occurring MDD and/or PTSD? and (2) what were the characteristics of the adaptations, in terms of key features of intervention content, context, and support activities?

Our approach was guided by two conceptual frameworks related to adaptation of evidence-based practices. First, the Map of Adaptation Process (MAP) provides a useful guide for adaptation across five steps: (a) assess the context of the target population, (b) select intervention to be adapted, (c) adapt the intervention iteratively, (d) rigorously test the adapted intervention for effectiveness, and (e) implement the adapted intervention if effectiveness is confirmed [1]. By following this process, intervention developers and their community partners can systematically incorporate and test a variety of adaptations most likely to be necessary or beneficial. The second framework helps to document adaptations systematically, detailing features of the adapted intervention and providing insights into future adaptations of the original evidence-based practice (or others with similar features). The Framework for Reporting Adaptations and Modifications-Enhanced [2], distinguishes among several key types of adaptations: intervention content (e.g., adding or removing elements, shortening/lengthening pacing), context (e.g., changes in format, setting, or personnel), and support activities (e.g., changes to training, implementation, or evaluation). FRAME is a coding framework that captures the process and rationale behind adaptations to an intervention. Process codes describe the steps taken during adaptation, and rationale codes describe what factors the adaptation was meant to address. Based on these frameworks, we understood that an integrated collaborative care model for OUD with MDD and/or PTSD would require adaptations that incorporate features of disorder-specific treatment models and unique considerations for co-occurring disorders, while still preserving the core components of the collaborative care model.

## Methods

### Contextual information about the study

Our adapted collaborative care model is called CLARO, which stands for Collaboration Leading to Addiction Treatment and Recovery from Other Stresses. CLARO means “clear” in Spanish and is used as a word of affirmation (e.g., “¡Claro que sí!”). We adapted CLARO to provide a team-based primary care intervention for patients with OUD and co-occurring MDD and/or PTSD that was grounded in the evidence base of the collaborative care model.

The project is led by a partnership between the RAND Corporation, University of New Mexico, and Boston Medical Center along with 14 primary care clinics in New Mexico. The primary care partners include 11 clinics from two different Federally Qualified Health Centers (8 from one system, 3 from the other), plus 3 University of New Mexico health system clinics. Eight clinics are clustered centrally in Albuquerque near the University of New Mexico, and the remaining six clinics are in rural areas outside the city and in southern New Mexico.

New Mexico is a state with high levels of need. In 2019, New Mexico had the 12th highest drug overdose rate in the U.S and two-thirds of those deaths were opioid-related. About 49% of the state’s population is Hispanic [45] and Spanish is widely spoken. In addition, New Mexico is a state with high poverty rates [46], and high rates of death by suicide and drug overdose [41]. Most of the state is rural and Hispanic, and nearly every county in New Mexico is designated in a health professional shortage area. Thus, our adaptation processes needed to consider an intervention that would work within the context of New Mexico community-based primary care, in addition to considerations around the clinical population targeted by CLARO.

### Adaptation processes

**Fig. 1:**
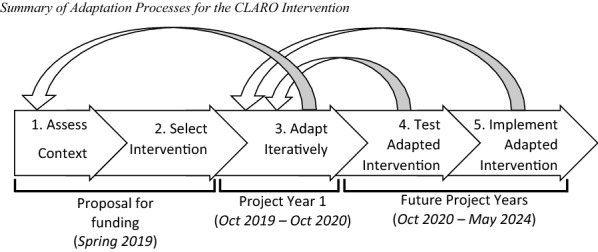
Summary of Adaptation Processes for the CLARO Intervention. CLARO: Collaboration Leading to Addiction Treatment and Recovery from Other Stresses. The five steps shown are from the Map of Adaptation Process [1]. Recursive arrows indicate the potential to revisit earlier steps as adaptation proceeds

Figure 1 provides a summary of the five MAP steps and shows how they align with the project timeline. Step 1 (assess context) and Step 2 (select intervention) took place in Spring 2019 during the proposal process for our Cooperative Agreement with the National Institute of Mental Health to develop and test CLARO. We then completed Step 3 (adapt iteratively) in the first year of the project, from October 2019 through October 2020. This paper focuses on Steps 1 through 3; we will discuss our plans for completing Step 4 (test adapted intervention) and Step 5 (implement adapted intervention) when we describe future directions for the project, which continues through May 2024. It should be noted that MAP emphasizes revisiting earlier steps as needed throughout adaptation, so we have refined our understanding of context throughout the project (revisiting Step 1) and we anticipate making additional adaptations while testing and implementing CLARO in our randomized trial (revisiting Step 3).

Table 1 summarizes the specific methods we used to complete the first three steps of MAP for CLARO. This project exemplifies participatory research, in which the intended target population and stakeholders of research are included in the process [47]; participatory research is also a foundation of the MAP [1]. Thus, for each MAP step, our team of research and clinical experts has worked collaboratively with the leadership of our primary health system partners, including executives (e.g., CEOs, COOs); directors of medical and behavioral health services; and clinic administrators (e.g., office managers, OUD program managers). Our partnership with these stakeholders began in the proposal-writing phase when they helped us assess the context of the target population (patients with OUD and co-occurring MDD/PTSD with few resources available to them) and select the intervention to be adapted (collaborative care). According to MAP Step 1, assessing context involves considering the characteristics of the target population (e.g., risk factors, community trends, accessibility considerations), organizational and stakeholder contexts (e.g., available resources, partnerships, experience with target population), and available interventions (e.g., core elements, resource requirements, theories of change). Triangulation of goodness-of-fit among the target population, context, and interventions leads to the ultimate decision of which intervention to adapt in MAP Step 2.

**Table 1:** Summary of methods used in Map of Adaptation Process for the CLARO Intervention

MAP Step	When	Who involved	Methods used
Step 1: Assess the Context of the Target Population	Proposal phase (Spring 2019)	Research team Clinic leadership	Identify goodness-of-fit considerations for target population, stakeholders, organizations, and interventions
Step 2: Select Intervention to be Adapted	Proposal phase (Spring 2019)	Research team Clinic leadership	Select intervention that best addresses triangulated Step 1 goodness-of-fit considerations
Step 3: Adapt the Intervention Iteratively	Project Year 1 (October 2019 to October 2020)	CLARO Adaptation Team Clinic patients Research Advisory Board Clinic leadership	Adapt, pre-test, and prepare to deliver the intervention. This included the following activities: Adaptation team meetings and review of materials Patient interviews Patient beta-testing Research Advisory Board meetings Clinic leadership meetings

CLARO: Collaboration Leading to Addiction Treatment and Recovery from Other Stresses. The three steps listed are from the Map of Adaptation Process [1]

MAP Step 3 involves making needed adaptations to the intervention, while seeking to preserve its core elements as much as possible; pre-testing the adaptations with members of the target population, and modifying adaptations iteratively as needed; and preparing for large-scale delivery and testing of the adapted intervention. Selection of CLARO adaptations was overseen by our Adaptation Team, a subgroup of the overall research team with expertise in OUD, MDD, and PTSD treatment in primary care; collaborative care models; implementation and adaptation of evidence-based practices, and community health settings. The Adaptation Team maintained engagement with health system stakeholders, and also received feedback from patients at the clinics and from our Research Advisory Board (a group of seven state and national experts in care for opioid use disorder and co-occurring disorders). The Adaptation Team met weekly throughout our year-long Step 3 adaptation period, during which time the team discussed feedback and potential adaptations; reviewed adapted materials developed by team members; and made (and documented) adaptation decisions for CLARO.

### Participants and interview procedures

During Step 3 (adapt iteratively), we engaged 11 patients (of 13 nominated by clinic providers) from the partner clinics in two rounds of interviews. While this sample size is consistent with prior qualitative work, our goal was to reach saturation with the information drawn whereby new insights were exhausted and no additional interviews were needed [48].

The first round consisted of informational interviews with 11 patients prior to intervention adaptation and the second round of interviews involved beta-testing of an initial CLARO visit followed by a debriefing interview (with 9 of the same patients). Thus, there were a total of 20 interviews conducted. This procedure was designated human subjects research and approved by the RAND Human Subjects Protection Committee (Protocol #2019-0509).

Clinic providers nominated patients with OUD and co-occurring MDD and/or PTSD to participate in the interviews. If a patient was interested, the provider completed a consent-to-contact form and forwarded the information to RAND to describe and schedule the interview. Interviews were conducted by phone in English and took about an hour to complete. We aimed to sample patients from each of the three participating health systems and while we had a bilingual staff member to conduct interviews in Spanish, all 11 patients were proficient in English. Participants were on average 52.4 (SD = 9.5) years old, 72.8% (n = 8) female, and 55% (n = 6) Hispanic. All interviews were audio recorded for reference during data analysis.

We conducted two rounds of interviews. In the first set of interviews, we gathered feedback from patients on topics related to experiences with OUD, MDD, and/or PTSD; experiences with medication and behavioral treatments for these diagnoses; and thoughts on how a Care Coordinator could improve patient engagement. A research assistant was present to take detailed field notes while the interviewer and patient talked. In the second round near the end of Step 3, we beta-tested a collaborative care session in which the interviewer role-played an initial visit with the patient. After beta-testing, a research assistant interviewed the patient one-on-one (again taking detailed field notes) and discussed the patient’s reactions to the session and suggestions for improvement; similarities and differences from existing services; and factors that might impact patient responsiveness to the CLARO intervention.

The interviewers and research assistants used a process of rapid content analysis to identify interview themes [49] so that the themes could be immediately incorporated into refinements of CLARO adaptations. After completing each round of interviews, three coders separately reviewed the interview recordings and notes. The purpose of the review was to identify themes related to the intervention’s feasibility and acceptability, including recommendations for the intervention. As a group, the coding team discussed preliminary themes they had identified from the data to create a unified list. The coders then independently reviewed transcripts again, and coded passages that were representative of each theme [50, 51]. The coding team met a second time to reconcile any coding discrepancies and achieve consensus on the themes, making revisions to the themes as needed.

### Documentation of adaptations

**Fig. 2:**
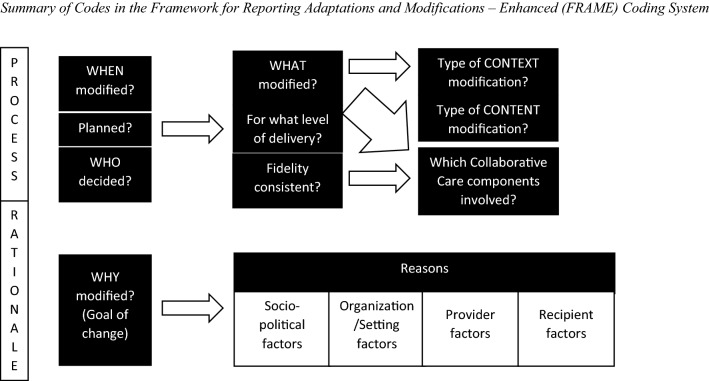
Summary of Codes in the Framework for Reporting Adaptations and Modifications – Enhanced (FRAME) Coding System. This figure is adapted from Fig. 1 in Wiltsey-Stirman et al. [2]. The original article was distributed under the terms of the Creative Commons Attribution 4.0 International License (http://creativecommons.org/licenses/by/4.0/), which permits unrestricted use, distribution, and reproduction in any medium, provided appropriate credit is given and any changes are noted. Our figure is a simplified version of the original, and our figure includes a new code we added for this project (Collaborative Care components)

The MAP recommends documenting adaptation decisions as they occur [1]. Our team used FRAME to document each adaptation we made to the CLARO intervention in Step 3. Figure 2 presents an overview of the FRAME coding system, in which each box represents a piece of information to be coded. To support our coding process, we created an Excel coding template that lists each FRAME code, with drop-down menus and space to enter additional details when needed; instructions for each code, taken from the FRAME Coding Manual [52], are included on separate tabs. The FRAME developers were not involved in creating this coding template, but they have reviewed it and posted it on their website, indicating their approval of its accuracy and usefulness [53].

The FRAME Coding Manual [52] describes each code in detail; they are summarized here. Process codes include *when* the adaptation occurred (e.g., pre-implementation, implementation, sustainment); whether the adaptation was *planned* (e.g., proactive vs. reactive); *who decided* (including all those involved and who made the ultimate adaptation decision); *what* was modified (i.e., the intervention’s content, the context in which it is delivered, training/evaluation plans, or implementation/scale-up activities) and at what *level of delivery* (e.g., for the target intervention group, a certain site/context, or for individual providers/recipients); and whether the adaptation was *fidelity-consistent*. For content and context adaptation, there are additional codes for specifying the *type of content modification* (e.g., adding or removing elements, shortening/lengthening pacing) or *type of context modification* (e.g., changes in format, setting, or personnel). For this project, we added a new code capturing which *components of the collaborative care model* were involved in the adaptation; we based the coding options on the seven core components of collaborative care [54] mentioned previously (e.g., team communication and care coordination, providing evidence-based treatment, systematic population-based management and follow-up). This code helped us expand on our understanding of intervention fidelity beyond whether the adaptations were fidelity-consistent or not. Finally, the FRAME rationale codes include the *goal* of the adaptation (e.g., improve fit, increase retention; we added a code option here, “response to COVID-19”), and note specific *reasons for the adaptation* from an extensive list of multi-level social-ecological drivers. Possible reasons can include sociopolitical factors (e.g., existing policies, societal/cultural norms, funding), organizational setting factors (e.g., available resources, competing demands, organizational culture), provider factors (e.g., training and skills, perceptions of the intervention), and recipient factors (e.g., cultural identities and beliefs, legal status, education level).

Each week when the CLARO Adaptation Team met, the second author used the FRAME coding template to capture information about any adaptations that were discussed, thus documenting the team’s adaptation decisions. The first author regularly reviewed the coding template and provided feedback. Any discrepancies were discussed with the full Adaptation Team to reach consensus about which adaptations were documented and using which codes.

## Results

We summarize results using the first three MAP steps and then summarize 12 adaptations we made in developing the CLARO intervention. Results from MAP Steps 1 and 2 were early intervention adaptations conducted at the proposal stage, while MAP Step 3 were late intervention adaptations to the intervention once the study was funded.

### MAP Step 1: assess context with stakeholders

**Fig. 3:**
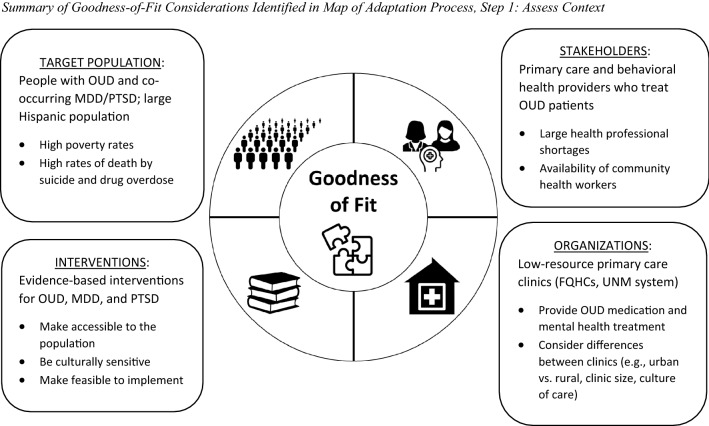
Overview of the Adapted CLARO Collaborative Care Model. CLARO: Collaboration Leading to Addiction Treatment and Recovery from Other Stresses; CC: Care Coordinator. Figure is adapted from the NIH Helping to End Addiction Long-Term (HEAL) Initiative^SM^

Figure 3 summarizes the considerations our team and partners identified for goodness of fit across target population, stakeholders, organizations, and interventions.

#### Target population

Our target population receiving the CLARO intervention was patients with opioid use disorder and co-occurring MDD and/or PTSD. Understanding the context in which these patients were living was important, especially because of their extensive social needs and because our clinic partners expressed concerns about the adequacy of standard OUD care for these patients.

#### Stakeholders

We also considered the skills, needs, and availability of various clinic stakeholders, including primary care and behavioral health providers. Clinics had existing OUD-specific programs for patients; however, like most community health centers, providers had limited capacity for managing complex patients. Implementing a traditional collaborative care model in which the care coordinators were nurses, social workers, or other licensed professionals was not feasible due to profound health professional shortages in New Mexico [55]. Instead, the state had an extensive history of using community health workers to enhance patient care and identified the use of these staff to mitigate provider constraints.

#### Organizations

We identified and partnered with primary care clinics that were already reaching our target population. All were primarily serving patients with Medicaid insurance or who were uninsured. The partner clinics had limited budgets and infrastructure to support a new intervention. We also recognized the need for flexibility to accommodate important differences among the clinics (e.g., urban vs. rural location, clinic size, administrator-led vs. clinician-led culture of care).

#### Interventions

Given these contextual factors, we considered which interventions could best address the target problems of OUD, MDD, and PTSD. In addition to being evidence-based, we recognized patients would only benefit from interventions that were also (a) feasible for the clinics to implement and deliver, (b) accessible, and (c) responsive to the patients’ and clinics’ socio-cultural contexts.

### MAP Step 2: select intervention to adapt

Based on our context assessment, the CLARO team and stakeholders from our partner health systems decided to pursue NIH HEAL funding to adapt and test the collaborative care model. There was consensus that collaborative care would be a useful service delivery intervention for addressing opioid use disorder, MDD, and PTSD in New Mexico primary care clinics. The model is grounded in core principles rather than rigidly specified protocols, which we expected would promote the flexibility needed to account for important socio-cultural factors. Furthermore, grant funding would provide the resources needed for successful adaptation. This approach resulted in the CLARO project proposal that was funded in October 2019.

### MAP Step 3: iteratively adapt collaborative care

Step 3 involved making context-driven adaptations to collaborative care, pre-testing those adaptations, and preparing our clinic partners to implement the adapted CLARO model. All activities were mutually informative and iterative, rather than sequential, and were guided by our Adaptation Team.

#### Adaptation and pre-testing

Our patient interviews helped identify important considerations for adapting the collaborative care model into CLARO, as well as those key aspects of the original model that should be preserved. The major themes identified in the patient interviews were that patients valued a strong connection with their OUD medical provider; important barriers to treatment included stigma toward OUD and mental disorders, financial barriers, and treatment accessibility. The proposed intervention content (e.g., addressing co-occurring disorders) and format (e.g., phone visits) were seen as acceptable and beneficial. Overall, there was support for the proposed care coordinator role, with important caveats about the need to respect patient autonomy, build trust, and provide support early in treatment. The adaptation team developed and documented specific adaptations based on the results of the patient interviews, along with input from the Research Advisory Board. The Adaptation Team developed each adaptation and documented it within the CLARO intervention manual and training materials.

### Documented adaptations

We used FRAME codes to document 12 identified adaptations to the collaborative care model for CLARO. Table 2 provides a detailed list of the FRAME codes for each identified adaptation, and Table 3 summarizes the codes.

**Table 2:** Documented CLARO adaptations with associated FRAME codes

#	Adaptation description	Process codes			
		When	Planned?	Who decided	Ultimate decision
1	Use of consultant team to support Care Coordinator	Pre-implementation	Planned/Proactive	CLARO team	CLARO team
				Clinic administrators	
2	Community Health Workers performing Care Coordinator role and referring to other providers for treatment as needed	Pre-implementation	Planned/Proactive	CLARO team	CLARO team
				Clinic administrators	
3	Addition of Written Exposure Therapy and medication for PTSD, and medication treatment for OUD	Pre-implementation	Planned/Proactive	CLARO team	CLARO team
				Clinic administrators	
				Funder (NIMH)	
4	Development and use of standardized measure to track OUD symptoms	Pre-implementation	Planned/Proactive	CLARO team	CLARO team
				Clinic administrators	
5	Addition of measure to track PTSD symptoms, the PCL-5 (PTSD Checklist for DSM-5)	Pre-implementation	Planned/Proactive	CLARO team	CLARO team
				Clinic administrators	
6	Screening patients for social needs, and referring them to local resources as needed	Pre-implementation	Planned/Proactive	CLARO team	CLARO team
				Clinic administrators	

Process codes						
#	What modified	Level of delivery	Context modifications	Content modifications	Fidelity consistent?	Collaborative care components
1	Context	Target population	Personnel	n/a	Yes	Use of population-based registry
	Training/Evaluation		Population			Psychiatric case review
						Program oversight/improvement
2	Context	System/Community	Personnel	n/a	Yes	Patient identification and diagnosis
	Training/Evaluation					Engage in integrated care program
						Provide evidence-based treatment
						Team communication/coordination
3	Content	Target population	Population	Integrating another treatment	Yes	Provide evidence-based treatment
	Context					
4	Content	Target population	Population	Adding elements/modules	Yes	Patient identification and diagnosis
	Context					Use of population-based registry
5	Content	Target population	Population	Adding elements/modules	Yes	Patient identification and diagnosis
	Context					Use of population-based registry
6	Content	System/Community	n/a	Adding elements/modules	Yes	Team communication/coordination

Rationale codes					
#	Goals of modification	Sociopolitical factors	Organization/Setting factors	Provider factors	Recipient factors
1	Improve outcomes	–	Available resources	Previous training/skills	Comorbidity
	Improve feasibility				
2	Improve feasibility	Funding/resource availability	Available resources	–	–
			Social context		
3	Improve outcomes	–	–	–	Comorbidity
4	Increase engagement	–	–	–	Comorbidity
	Improve outcomes				
5	Improve outcomes	–	–	–	Comorbidity
6	Increase engagement	–	Available resources	Previous training/skills	Access to resources
	Increase retention				Crisis/emergency
	Increase satisfaction				

#	Adaptation description	Process codes			
		When	Planned?	Who decided	Ultimate decision
7	Engaging in additional outreach activities (e.g., home visits, attend social service appts)	Pre-implementation	Planned/Proactive	CLARO team	CLARO team
				Clinic administrators	
8	Use of interactive, practice-oriented training and ECHO for Care Coordinators; reflective supervision	Pre-implementation	Planned/Proactive	CLARO team	CLARO team
				Clinic administrators	
9	Addition of co-occurring disorders to Written Exposure Therapy and Problem-Solving Treatment trainings	Pre-implementation	Planned/Proactive	Treatment developer/ trainer	Treatment developer/trainer
				CLARO team	
10	Expanding patient registry to track progress in OUD, PTSD treatment	Pre-implementation	Planned/Proactive	Treatment developer/ trainer	CLARO team
				CLARO team	
11	Delivery of Problem-Solving Treatment training in virtual, video-conferencing format	Implementation	Planned/Reactive	Treatment developer/ trainer CLARO team Clinic administrators	Treatment developer/trainer
12	Delivery of Care Coordinator training in virtual, video-conferencing format	Implementation	Planned/Reactive	CLARO team	CLARO team

#	Process codes					
	What modified	Level of delivery	Context modifications	Content modifications	Fidelity consistent?	Collaborative care components
7	Context	System/Community	Setting	n/a	Yes	Engage in integrated care program
						Use of population-based registry
8	Context	System/Community	Personnel	n/a	Yes	Program oversight/improvement
	Training/Evaluation					
9	Context	Target population	Population	n/a	Yes	Provide evidence-based treatment
	Training/Evaluation					
10	Context	Target population	Population	n/a	Yes	Use of population-based registry
	Implementation					
11	Training/Evaluation	Cohort	n/a	n/a	Yes	Provide evidence-based treatment
						Program oversight/improvement
12	Training/Evaluation	Cohort	n/a	n/a	Yes	Provide evidence-based treatment
						Program oversight/improvement

	Rationale codes				
#	Goals of modification	Sociopolitical factors	Organization/Setting factors	Provider factors	Recipient factors
7	Increase engagement	–	Available resources	Previous training/skills	Access to resources
	Increase retention		Location/accessibility		Crisis/emergency
	Increase satisfaction				Motivation/readiness
8	Improve feasibility	–	Available resources	Previous training/skills	–
	Reduce cost		Social context		
9	Improve outcomes	–	–	–	Comorbidity
10	Improve outcomes	–	–	–	Comorbidity
11	Response to COVID-19	–	–	–	–
12	Response to COVID-19	–	–	–	–

Codes are based on the Framework for Reporting Adaptations and Modifications–Enhanced (FRAME; [2]) and the associated Coding Manual [52]. “n/a” indicates that the code was not applicable, because the adaptation did not fall within that category. “–" indicates that the rationale for the adaptation did not include factors from that social-ecological level. CLARO: Collaboration Leading to Addiction Treatment and Recovery from Other Stresses; OUD: opioid use disorder; MDD: major depressive disorder; PTSD: post-traumatic stress disorder; NIMH: National Institute of Mental Health; ECHO: Extension for Community Healthcare Outcomes

**Table 3:** Summary of FRAME codes for twelve documented CLARO adaptations

FRAME code	Code value	Number of adaptations	Percentage of adaptations
When			
	Pre-implementation	10	83%
	Implementation	2	17%
Planned?			
	Planned/Proactive	10	83%
	Planned/Reactive	2	17%
Who decided^a^			
	CLARO team	12	100%
	Clinic administrators	9	75%
	Treatment developer/trainer	3	25%
	Funder	1	8%
What modified^a^			
	Context	9	75%
	Content	4	33%
	Training/Evaluation	6	50%
	Implementation	1	8%
Level of delivery			
	Target population	6	50%
	System/Community	4	33%
	Cohort	2	17%
Context modifications^a^			
	Population	6	50%
	Personnel	3	25%
	Setting	1	8%
	n/a	3	25%
Content modifications			
	Adding elements/modules	3	25%
	Integrating another treatment	1	8%
	n/a	8	67%
Fidelity consistent?			
	Yes	12	100%
Collaborative care components^a^			
	Patient identification and diagnosis	3	25%
	Engage in integrated care program	2	17%
	Provide evidence-based treatment	5	42%
	Use of population-based registry	5	42%
	Team communication/coordination	2	17
	Psychiatric case review	1	8
	Program oversight/improvement	4	33
Goals of modification^a^			
	Increase engagement	3	25
	Increase retention	2	17
	Improve feasibility	3	25
	Improve outcomes	6	50
	Reduce cost	1	8
	Increase satisfaction	2	17
	Response to COVID-19	2	17
Sociopolitical factors^b^			
	Funding/resource availability	1	8
Organization/Setting factors^b^			
	Available resources	5	42
	Location/accessibility	1	8
	Social context	2	17
Provider factors^b^			
	Previous training/skills	4	33
Recipient factors^b^			
	Access to resources	2	17
	Comorbidity	6	50
	Crisis/emergency	2	17
	Motivation/readiness	1	8

Codes are based on the Framework for Reporting Adaptations and Modifications–Enhanced (FRAME) and the associated Coding Manual [2, 52]. CLARO: :Collaboration Leading to Addiction Treatment and Recovery from Other Stresses

^a^More than one code value can apply to the same adaptation, so the percentages of adaptations for this code sum to greater than 100%

^b^Each adaptation is assigned values from across these four rationale codes, so the percentages of adaptations do not necessarily sum to 100%

In terms of process, most adaptations were made pre-implementation and were planned/proactive (83% each). The two planned/reactive adaptations during implementation both involved changing to virtual trainings in response to COVID-19. The CLARO team was involved in 100% of adaptation decisions and made the final decision on most (83%) but treatment developers/trainers made the final decision on two adaptations to their trainings (17%). The most common targets for adaptation were the intervention context (75%) and training/evaluation (50%), but we also made content (33%) and implementation/scale-up (8%) adaptations. Adaptations were most often made at the level of all treatment recipients (i.e., patients with OUD and co-occurring MDD/PTSD; 50%) or the health system/community (i.e., New Mexico primary care clinics; 33%), but the two responses to the COVID-19 pandemic were just for the initial training cohorts. Context modifications most often involved adapting collaborative care for the new target population and personnel (i.e., community health workers hired as CCs); all content modifications involved adding elements/modules or integrating another treatment (i.e., we only used two of the 15 possible content codes). Finally, the Adaptation team reviewed all adaptations to make sure the adaptations were fidelity-consistent, thus maintaining the core components of collaborative care. All core principles of collaborative care [34] were involved in one or more of the adaptations, most commonly provision of evidence-based treatment and use of the population-based registry for follow-up and treatment adjustment (42% each), and least commonly psychiatric case review (only one adaptation).

Adaptations were most commonly made with the goal of improving outcomes (50%) and increasing engagement or feasibility (25% each). There were also adaptations aimed at increasing retention, reducing cost, and increasing satisfaction. Two adaptations were made in response to COVID-19. In terms of the social-ecological factors that drove the adaptation decisions, the most common (50%) was addressing intervention recipients’ comorbidity (i.e., co-occurrence of OUD with MDD/PTSD). Overall, recipient characteristics were part of the rationale for 67% of all adaptations. Other factors related to the CLARO adaptations were noted at the sociopolitical level (8%; funding/resource availability only); the organization/setting level (42%; most common factor was available resources); and the provider level (33%; previous training/skills only).

### CLARO collaborative care model

**Fig. 4:**
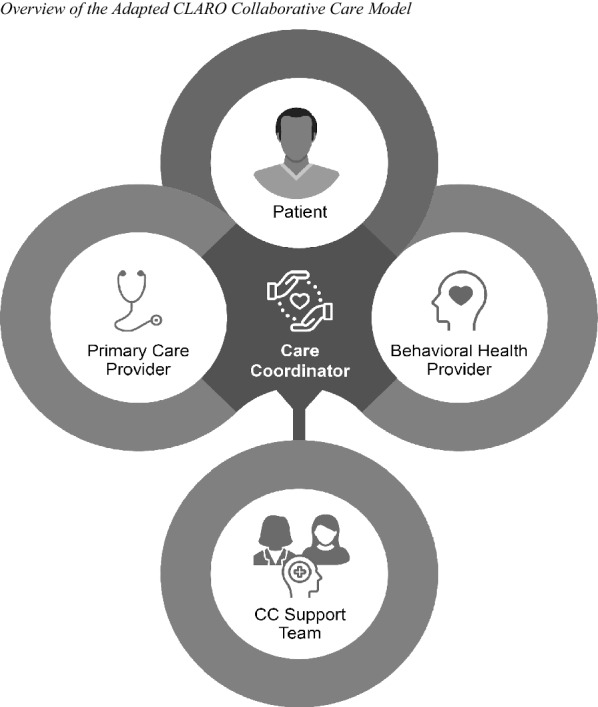
Summary of Goodness-of-Fit Considerations Identified in Map of Adaptation Process, Step 1: Assess Context. Based on the Map of Adaptation Process [1]. OUD: opioid use disorder; MDD: major depressive disorder; PTSD: post-traumatic stress disorder; FQHC: Federally Qualified Health Center; UNM: University of New Mexico

In Fig. 4, we summarize the adapted CLARO model to explain how the previously described adaptations fit together. The care coordinator is at the center of the model, which is fidelity-consistent, but use of Community Health Workers was a major adaptation to fit the health professional shortage in New Mexico. Community Health Workers are lay professionals, often with a high school or college education, who come from the community being served and provide a bridge to care for often underserved, underrepresented populations [56]. They are also adept at addressing social needs such as housing, transportation, and caregiving needs, which can become barriers to care.

We developed a detailed intervention manual and 24 h of training for care coordinators across 2 weeks. We specified a visit schedule for Care Coordinators to meet regularly with patients over 13 visits across a six-month intervention period, starting with more frequent visits and decreasing visit frequency over time. Care coordinators were also given access to a web-based registry that was specifically designed for managing and tracking CLARO patient progress and caseload. Finally, care coordinators were given support from a supervision team, including a psychiatric consultant to review all of their cases, a consultant who is a Master Community Health Worker to provide coaching in how to integrate with their care team and address patients’ social needs, and two CLARO supervisors to assist with day-to-day tasks and monitor CLARO model fidelity. We established a schedule of 3 h of supervision meetings per week in various forms, ranging from individual to group meetings. These types of support were seen as essential to promote success when expanding the scope of Community Health Workers’ responsibilities for the care coordinator role.

Standard of care for these clinics was to offer buprenorphine/naloxone for OUD and medication treatment for MDD and PTSD. Patients who previously did not engage with buprenorphine treatment were offered a community referral to intensive outpatient treatment or methadone maintenance. The study team trained the clinic’s mental health clinicians in two evidence-based psychotherapies; providers were encouraged to offer these treatments to any patient who could benefit from them. Problem-Solving Therapy for MDD involves teaching skills for identifying and solving problems in everyday life to improve mood and behavioral activation [57] and has been used in collaborative care previously. Written exposure therapy for PTSD involves use of a written trauma narrative to facilitate exposure and processing of traumatic event memories [58], and was a novel treatment to use within a collaborative care model. The trainings provided to behavioral health providers at each clinic were modified to better address co-occurring opioid use (e.g., through case vignettes). Care coordinators were tasked with helping patients think through treatment options and initiate desired treatments, as well as monthly administration of evidence-based measures that track patient progress on opioid use and depression and/or PTSD (e.g., [59–61]), as relevant. 

## Discussion

This study adds to the knowledge base on collaborative care by describing our adaptation processes for implementing collaborative care for co-occurring OUD and mental health disorders in a low-resource state. We extended the literature by using MAP to guide our early and late intervention adaptation activities and the FRAME to track our adaptations. In doing so, this study contributed important advances in the use of implementation science to adapt evidence-based interventions. Documentation of our adaptation processes and outcomes provided key insights into how treatment developers can systematically (a) make adaptations to evidence-based practices while (b) understanding the implications of what was adapted and how, while testing the effectiveness of those adaptations.

We adapted our CLARO collaborative care intervention to utilize Community Health Workers as care coordinators and built in a greater focus on addressing patients’ social needs. We supported care coordinators by implementing a supervision team to support their training needs, and also modified the training protocols for Problem-Solving Therapy and Written Exposure Therapy to address co-occurring OUD. We described an alternate approach to the collaborative care intervention through Community Health Workers that could prove more broadly scalable and sustainable than other approaches that rely on more specialized healthcare providers in the care coordinator role (e.g., behavioral health, nursing).

Implementation science has recently begun placing long-overdue emphasis on sustainability, defined as long-term maintenance of an evidence-based practice as part of routine services after implementation is complete [62, 63]. Nationally, uptake of collaborative care in primary care settings has been slow—even with Centers for Medicare and Medicaid Services offering dedicated billing codes—in part because the available reimbursement does not appear to sustainably cover the ongoing expenses of specialized providers delivering care coordination [64, 65]. We plan to explore sustainability considerations for the CLARO intervention with our healthcare system partners following the trial, once they are able to make an informed decision on whether they wish to sustain the adapted CLARO intervention and what the associated costs would be. We have also begun informal conversations with New Mexico policymakers about the possibility of activating dedicated collaborative care billing codes for the state’s Medicaid program.

We sought to bring a high level of rigor to documenting our adaptation processes and outcomes by following the MAP [1] and using the FRAME [2] to code adaptations. Both MAP and FRAME have been cited in hundreds of publications, but few researchers have reported a detailed step-by-step application of these frameworks with other evidence-based practices (for rare examples, see [66, 67]). More systematic application of these frameworks is needed to advance the science and practice of adapting evidence-based models, and to illustrate their use with diverse types of practices and settings. Rigor in this area of research could be further enhanced by detailed guidance on selecting an adaptation framework from the many options available; we chose MAP due to its comprehensiveness and widespread recognition, but comparison with alternative frameworks was difficult. It would be useful to expand available tools for selection of implementation frameworks [68, 69] to include adaptation frameworks. For coding adaptations, on the other hand, FRAME is the only comprehensive guidance available—so the main issue was its utility, which we generally found to be high. We did seek to improve the usability of the FRAME by developing our Excel coding template, which is now available on the FRAME developers’ website. We also found our addition of a new code (which intervention components involved) to be a useful contribution to the FRAME, because it provides additional context for the Yes/No code stating whether an adaptation was fidelity-consistent; given that successful implementation often requires flexibility within fidelity [70], we found a binary code insufficient for this purpose. Overall, the science of adapting evidence-based practices is still developing and is ripe with future directions to explore.

This study is limited in several ways. First, we only documented adaptations during the proposal and intervention preparatory stages prior to the start of our randomized controlled trial.

Since completing this analysis, we have begun documenting more reactive adaptations, and we anticipate that certain clinics or care coordinators could make unplanned adaptations as well. Second, we did not use data on implementation, service, or patient outcomes to select adaptations. Our input from stakeholders and discussions within our Adaptation team improved the overall feasibility of the adaptation approach, but not using other data sources makes it difficult to distinguish which adaptations were essential versus optional versus benign but ineffective (harmful adaptations are possible as well, but much more likely to be identified through our approach). Third, we did not provide a detailed description of the themes from our rapid content analysis of patient interviews in this article, but such rich detail could be useful for more fully understanding adaptation decisions. We plan to publish the qualitative themes separately so they can be presented as fully as possible. Finally, our qualitative data were inclusive of a small sample of patient interviews and other stakeholder input, and a greater sample size might have elicited more information that could inform adaptation and/or more “thick description” of adaptations and their rationale (as a complement to the highly structured FRAME codes).

Our larger study is currently in Step 4 of MAP to continue tracking adaptations with the FRAME, both those proactively decided by our team and unplanned, reactive adaptations made by the clinics. The final MAP step is long-term and large-scale use of the adapted intervention, and we will help the clinics plan for sustaining CLARO beyond the study if they wish.

## Conclusion

An important aspect of adapting interventions is to systematically document decision-making to better understand why adaptations are being made and whether these adaptations are consistent with key intervention principles or components. This study documented 12 collaborative-care consistent adaptations, ten of which were made prior to implementing our trial and two in reaction to the COVID-19 pandemic. Major adaptations to reach this patient population and optimize service delivery in our clinics included use of a community health worker as care coordinators, a registry to track patient progress, and a robust care team to support care coordinators. We also added measurement-based care and brief therapies to directly address OUD, MDD, and PTSD symptomatology. Finally, to respond to the local context in New Mexico, we included measurement of social determinants of health and additional outreach activities such as home visits. In adapting collaborative care for CLARO, we aimed to respond to implementation barriers for reaching this patient population and to ultimately improve the service delivery for individuals experiencing the devastating consequences of co-occurring OUD with MDD/PTSD. Our findings can inform continued efforts to adapt the highly flexible collaborative care intervention to a variety of clinical populations and settings and may also serve as a blueprint for adaptation of other complex evidence-based practices in healthcare settings and beyond.

## Data Availability

The datasets used during the current study are available upon request through the corresponding author.

## Abbreviations

CC: Care coordinators
CLARO: Collaboration Leading to Addiction Treatment and Recovery from Other Stresses
COD: Co-occurring disorders
COVID: Coronavirus disease 2019
FRAME: Framework for Reporting Adaptations and Modifications–Enhanced
HEAL: Helping to End Addiction Long-term^SM^
MAP: Map of Adaptation Process
MDD: Major depressive disorder
MOUD: Medication for Opioid Use Disorder
NIH: National Institutes of Health
OUD: Opioid use disorder
PTSD: Post-traumatic stress disorder

## References

[1] McKleroy VS, Galbraith JS, Cummings B, Jones P, Harshbarger C, Collins C (2006). Adapting evidence–based behavioral interventions for new settings and target populations. AIDS Educ Prev.

[2] Wiltsey Stirman S, Baumann AA, Miller CJ (2019). The FRAME: an expanded framework for reporting adaptations and modifications to evidence-based interventions. Implement Sci.

[3] Barry DT, Cutter CJ, Beitel M, Kerns RD, Liong C, Schottenfeld RS (2016). Psychiatric disorders among patients seeking treatment for co-occurring chronic pain and opioid use disorder. J Clin Psychiatry.

[4] Compton WM, Thomas YF, Stinson FS, Grant BF (2007). Prevalence, correlates, disability, and comorbidity of DSM-IV drug abuse and dependence in the United States: results from the national epidemiologic survey on alcohol and related conditions. Arch Gen Psychiatry.

[5] Dore G, Mills K, Murray R, Teesson M, Farrugia P (2012). Post-traumatic stress disorder, depression and suicidality in inpatients with substance use disorders. Drug Alcohol Rev.

[6] Grant BF, Stinson FS, Dawson DA, Chou SP, Dufour MC, Compton W (2004). Prevalence and co-occurrence of substance use disorders and independent mood and anxiety disorders: results from the National Epidemiologic Survey on Alcohol and Related Conditions. Arch Gen Psychiatry.

[7] Han B, Compton WM, Blanco C, Colpe LJ (2017). Prevalence, treatment, and unmet treatment needs of US adults with mental health and substance use disorders. Health Aff.

[8] Meier A, Lambert-Harris C, McGovern MP, Xie H, An M, McLeman B (2014). Co-occurring prescription opioid use problems and posttraumatic stress disorder symptom severity. Am J Drug Alcohol Abuse.

[9] Mills KL, Teesson M, Ross J, Peters L (2006). Trauma, PTSD, and substance use disorders: findings from the Australian national survey of mental health and well-being. Am J Psychiatry.

[10] Brown PJ, Recupero PR, Stout R (1995). PTSD substance abuse comorbidity and treatment utilization. Addict Behav.

[11] Kessler RC, Sonnega A, Bromet E, Hughes M, Nelson CB (1995). Posttraumatic stress disorder in the National Comorbidity Survey. Arch Gen Psychiatry.

[12] Mills KL, Teesson M, Ross J, Darke S, Shanahan M (2005). The costs and outcomes of treatment for opioid dependence associated with posttraumatic stress disorder. Psychiatr Serv.

[13] Nunes EV, Sullivan MA, Levin FR (2004). Treatment of depression in patients with opiate dependence. Biol Psychiatry.

[14] Ouimette PC, Ahrens C, Moos RH, Finney JW (1997). Posttraumatic stress disorder in substance abuse patients: relationship to 1-year posttreatment outcomes. Psychol Addict Behav.

[15] Ouimette PC, Brown PJ, Najavits LM (1998). Course and treatment of patients with both substance use and posttraumatic stress disorders. Addict Behav.

[16] Fendrich M, Becker J, Hernandez-Meier J (2019). Psychiatric symptoms and recent overdose among people who use heroin or other opioids: results from a secondary analysis of an intervention study. Addict Behav Rep.

[17] Ilgen MA, Bohnert AS, Ignacio RV, McCarthy JF, Valenstein MM, Kim HM (2010). Psychiatric diagnoses and risk of suicide in veterans. Arch Gen Psychiatry.

[18] Jones CM, McCance-Katz EF (2019). Co-occurring substance use and mental disorders among adults with opioid use disorder. Drug Alcohol Depend.

[19] Mościcki E, O'Carroll P, Rae D, Locke B, Roy A, Regier D (1988). Suicide attempts in the Epidemiologic Catchment Area study. Yale J Biol Med.

[20] Darke S, Ross J, Marel C, Mills KL, Slade T, Burns L (2015). Patterns and correlates of attempted suicide amongst heroin users: 11-year follow-up of the Australian treatment outcome study cohort. Psychiatry Res.

[21] Ahmadi J, Jahromi MS, Ehsaei Z (2018). The effectiveness of different singly administered high doses of buprenorphine in reducing suicidal ideation in acutely depressed people with co-morbid opiate dependence: a randomized, double-blind, clinical trial. Trials.

[22] Gordon AJ, Lo-Ciganic W-H, Cochran G, Gellad WF, Cathers T, Kelley D (2015). Patterns and quality of buprenorphine opioid agonist treatment in a large Medicaid program. J Addict Med.

[23] Morgan JR, Schackman BR, Leff JA, Linas BP, Walley AY (2018). Injectable naltrexone, oral naltrexone, and buprenorphine utilization and discontinuation among individuals treated for opioid use disorder in a United States commercially insured population. J Subst Abuse Treat.

[24] Samples H, Williams AR, Olfson M, Crystal S (2018). Risk factors for discontinuation of buprenorphine treatment for opioid use disorders in a multi-state sample of Medicaid enrollees. J Subst Abuse Treat.

[25] Williams AR, Samples H, Crystal S, Olfson M (2020). Acute care, prescription opioid use, and overdose following discontinuation of long-term buprenorphine treatment for opioid use disorder. Am J Psychiatry.

[26] Harris KM, Edlund MJ (2005). Use of mental health care and substance abuse treatment among adults with co-occurring disorders. Psychiatr Serv.

[27] Priester MA, Browne T, Iachini A, Clone S, DeHart D, Seay KD (2016). Treatment access barriers and disparities among individuals with co-occurring mental health and substance use disorders: an integrative literature review. J Subst Abuse Treat.

[28] U.S. Department of Health and Human Services. Report to Congress on the prevention and treatment of co-occurring substance abuse disorders and mental disorders. Rockville: Substance Abuse and Mental Health Services Administration; 2002.

[29] Cherpitel CJ, Ye Y (2012). Trends in alcohol- and drug-related emergency department and primary care visits: data from four U.S. national surveys (1995–2010). J Stud Alcohol Drugs.

[30] Pilowsky DJ, Wu LT (2012). Screening for alcohol and drug use disorders among adults in primary care: a review. J Subst Abuse Rehabil.

[31] Alexander CL, Arnkoff DB, Glass CR (2010). Bringing psychotherapy to primary care: innovations and challenges. Clin Psychol Sci Pract.

[32] Beronio K, Po R, Skopec L. Affordable Care Act will expand mental health and substance use disorder benefits and parity protections for 62 million Americans. ASPE Research Brief. 2013. http://aspe.hhs.gov/health/reports/2013/mental/rb_mental.cfm. Accessed 20 Feb.

[33] Buck JA (2011). The looming expansion and transformation of public substance abuse treatment under the Affordable Care Act. Health Aff.

[34] University of Washington, Psychiatry & Behavioral Sciences, Division of Population Health, AIMS Center. Principles of collaborative care. 2019. https://aims.uw.edu/collaborative-care/principles-collaborative-care. Accessed 17 Mar 2020.

[35] Gilbody S, Bower P, Fletcher J, Richards D, Sutton AJ (2006). Collaborative care for depression: a cumulative meta-analysis and review of longer-term outcomes. Arch Intern Med.

[36] Meredith LS, Eisenman DP, Han B, Green BL, Kaltman S, Wong EC (2016). Impact of collaborative care for underserved patients with PTSD in primary care: a randomized controlled trial. J Gen Intern Med.

[37] Brackett CD, Duncan M, Wagner JF, Fineberg L, Kraft S (2021). Multidisciplinary treatment of opioid use disorder in primary care using the collaborative care model. Subst Abus.

[38] Watkins KE, Ober AJ, Lamp K, Lind M, Setodji C, Osilla KC (2017). Collaborative care for opioid and alcohol use disorders in primary care: The SUMMIT randomized clinical trial. JAMA Intern Med.

[39] Berenz EC, Coffey SF (2012). Treatment of co-occurring posttraumatic stress disorder and substance use disorders. Curr Psychiatry Rep.

[40] Meredith LS, Komaromy MS, Cefalu M, Murray-Krezan C, Page K, Osilla KC (2021). Design of CLARO (Collaboration Leading to Addiction Treatment and Recovery from other Stresses): A randomized trial of collaborative care for opioid use disorder and co-occurring depression and/or posttraumatic stress disorder. Contemp Clin Trials..

[41] New Mexico Department of Public Health. How New Mexico compares, 2019. 2019. https://ibis.health.state.nm.us/view/docs/HowNMCompares2019_2-pager.pdf. Accessed 2 July 2021.

[42] Damschroder LJ, Hagedorn HJ (2011). A guiding framework and approach for implementation research in substance use disorders treatment. Psychol Addict Behav.

[43] Baumann A, Cabassa L, Stirman S, Brownson R, Colditz G, Proctor E (2018). Adaptation in dissemination and implementation science. Dissemination and implementation research in health: translating science to practice.

[44] Castro F, Barrera JM, Holleran SL (2010). Issues and challenges in the design of culturally adapted evidence-based interventions. Annu Rev Clin Psychol.

[45] United States Census Bureau. QuickFacts: New Mexico. 2020. https://www.census.gov/quickfacts/NM. Accessed 2 July 2021.

[46] United States Census Bureau. American community survey (ACS). 2017. https://www.census.gov/programs-surveys/acs/about.html. Accessed 4 Mar 2019.

[47] Cargo M, Mercer SL (2008). The value and challenges of participatory research: strengthening its practice. Annu Rev Public Health.

[48] Hennink M, Kaiser BN (2022). Sample sizes for saturation in qualitative research: a systematic review of empirical tests. Soc Sci Med.

[49] McNall M, Foster-Fishman PG (2007). Methods of rapid evaluation, assessment, and appraisal. Am J Eval.

[50] Krippendorf K (1980). Content analysis: an introduction to its methodology.

[51] Weber RP (1990). Basic content analysis.

[52] Wiltsey Stirman S, Baumann AA, Miller CJ. FRAME coding manual. 2019. https://med.stanford.edu/fastlab/research/adaptation.html Accessed 2 July 2021.

[53] Stanford Medicine, The F.A.S.T. Lab. Adaptation: FRAME-IS for tracking implementation strategies. 2021. https://med.stanford.edu/fastlab/research/adaptation.html. Accessed 16 July 2021.

[54] University of Washington, Psychiatry & Behavioral Sciences, Division of Population Health, AIMS Center. Patient-centered integrated behavioral health care: Principles & tasks checklist. 2014. https://aims.uw.edu/sites/default/files/CollaborativeCarePrinciplesAndComponents_2014-12-23.pdf. Accessed 3 June 2020.

[55] Rural Health Information Hub. Health professional shortage areas: Primary care, by county, 2021 - New Mexico. 2020. https://www.ruralhealthinfo.org/charts/5?state=NM Accessed 2 July 2021.

[56] Hartzler AL, Tuzzio L, Hsu C, Wagner EH (2018). Roles and functions of community health workers in primary care. Ann Fam Med.

[57] Renn BN, Mosser BA, Raue PJ, Tampi RR, Yarns BC, Zdanys KF, Tampi DJ (2020). Problem-solving therapy. Psychotherapy in later life.

[58] Sloan DM, Marx BP (2019). Written Exposure Therapy for PTSD: a brief treatment approach for mental health professionals.

[59] Blevins CA, Weathers FW, Davis MT, Witte TK, Domino JL (2015). The Posttraumatic Stress Disorder Checklist for DSM-5 (PCL-5): development and initial psychometric evaluation. J Trauma Stress.

[60] Pilkonis PA, Yu L, Dodds NE, Johnston KL, Lawrence SM, Hilton TF (2015). Item banks for substance use from the Patient-Reported Outcomes Measurement Information System (PROMIS(®)): Severity of use and positive appeal of use. Drug Alcohol Depend.

[61] Spitzer RL, Kroenke K, Williams JB (1999). Validation and utility of a self-report version of PRIME-MD: The PHQ primary care study. Primary care evaluation of mental disorders Patient health questionnaire. JAMA.

[62] Proctor E, Luke D, Calhoun A, McMillen C, Brownson R, McCrary S (2015). Sustainability of evidence-based healthcare: research agenda, methodological advances, and infrastructure support. Implement Sci.

[63] Shelton RC, Lee M (2019). Sustaining evidence-based interventions and policies: recent innovations and future directions in implementation science. Am J Public Health.

[64] Carlo AD, Corage Baden A, McCarty RL, Ratzliff ADH (2019). Early health system experiences with collaborative care (CoCM) billing codes: a qualitative study of leadership and support staff. J Gen Intern Med.

[65] Carlo AD, Drake L, Ratzliff AD, Chang D, Unützer J (2020). Sustaining the collaborative care model (CoCM): billing newly available CoCM CPT codes in an academic primary care system. Psychiatr Serv.

[66] Chlebowski C, Hurwich-Reiss E, Wright B, Brookman-Frazee L (2020). Using stakeholder perspectives to guide systematic adaptation of an autism mental health intervention for Latinx families: a qualitative study. J Community Psychol.

[67] Poulsen MN, Vandenhoudt H, Wyckoff SC, Obong'o CO, Ochura J, Njika G (2010). Cultural adaptation of a US evidence-based parenting intervention for rural Western Kenya: from parents matter! To families matter!. AIDS Educ Prev.

[68] Birken SA, Rohweder CL, Powell BJ, Shea CM, Scott J, Leeman J (2018). T-CaST: an implementation theory comparison and selection tool. Implement Sci.

[69] Dissemination-implementation.org. Dissemination & implementation models in health research and practice. Undated. https://dissemination-implementation.org/content/diMain.aspx Accessed 11 Feb 2022.

[70] Kendall PC, Gosch E, Furr JM, Sood E (2008). Flexibility within fidelity. J Am Acad Child Adolesc Psychiatry.

